# Biomarkers of iron metabolism in chronic kidney disease

**DOI:** 10.1007/s11255-020-02663-z

**Published:** 2020-10-06

**Authors:** Glogowski Tomasz, Wojtaszek Ewa, Malyszko Jolanta

**Affiliations:** Department of Nephrology, Dialysis and Internal Medicine, Medical University of Warsaw, ul. Banacha 1a, 02-097 Warsaw, Poland

**Keywords:** Iron metabolism, Hepcidin, Chronic kidney disease

## Abstract

Iron is the most abundant transition metal in the human body and an essential element required for growth and survival. Our understanding of the molecular control of iron metabolism has increased dramatically over the past 20 years due to the discovery of hepcidin, which regulates the uptake of dietary iron and its mobilization from macrophages and hepatic stores. Anemia and iron deficiency are common in chronic kidney disease. The pathogenesis of anemia of chronic kidney disease is multifactorial. Correction of anemia requires two main treatment strategies: increased stimulation of erythropoiesis, and maintenance of an adequate iron supply to the bone marrow. However, there are still many uncertainties in regard to iron metabolism in patients with chronic kidney disease and in renal replacement therapy. The aim of this review was to summarize the current knowledge on iron metabolism in this population, including new biomarkers of iron status. There is an area of uncertainty regarding diagnostic utility of both erythroferrone (ERFE) and hepcidin in end-stage renal disease (ESRD) patients. Higher concentration of hepcidin in oligoanuric patients may reflect decreased renal clearance. Furthermore, the hepcidin-lowering effect of ERFE in ESRD patients treated with erythropoiesis-stimulating agents (ESAs) may be blunted by underlying inflammation and concomitant iron treatment. Thus, future studies should validate the use of ERFE as a biomarker of erythropoiesis and predictor of response to iron and ESA therapy in dialysis-dependent patients.

## Introduction

According to World Health Organization (WHO), anemia is defined as a hemoglobin concentration below 13 g/dl for adult males and below 12 g/dl for non-pregnant women [1]. The most common cause of anemia worldwide is iron deficiency, while anemia of inflammation is the second most prevalent type. Prevalence of anemia in patients with chronic kidney disease (CKD) increases in more advanced stages of CKD, affecting the majority of stage G4 patients (eGFR of 15 to 30 ml/min) [2, 3]. There are several underlying factors contributing to anemia in this population—relative erythropoietin deficiency, iron deficiency (both absolute and functional), impaired hepcidin clearance, shorter erythrocyte lifespan, and nutritional deficiencies (folic acid and vitamin B12, among others). CKD stage G5 patients on hemodialysis (HD) have additional iron loss (up to 3 g per year) [4] as a consequence of chronic bleeding, repeated phlebotomy (venipuncture) and blood lost in the dialyzer and the lines. Furthermore, both HD and peritoneal dialysis (PD) patients are likely to develop chronic subclinical inflammation as a result of exposure to dialyzer membrane and drains and non-biocompatible dialysis fluid, respectively. Anemia in CKD patients leads to reduced quality of life and cardiovascular performance, cognitive impairment, increased rate of hospitalizations and increased mortality [4]. Additionally, anemia may contribute to accelerated progression to end-stage renal disease (ESRD) [5].

## Iron metabolism

**Fig. 1:**
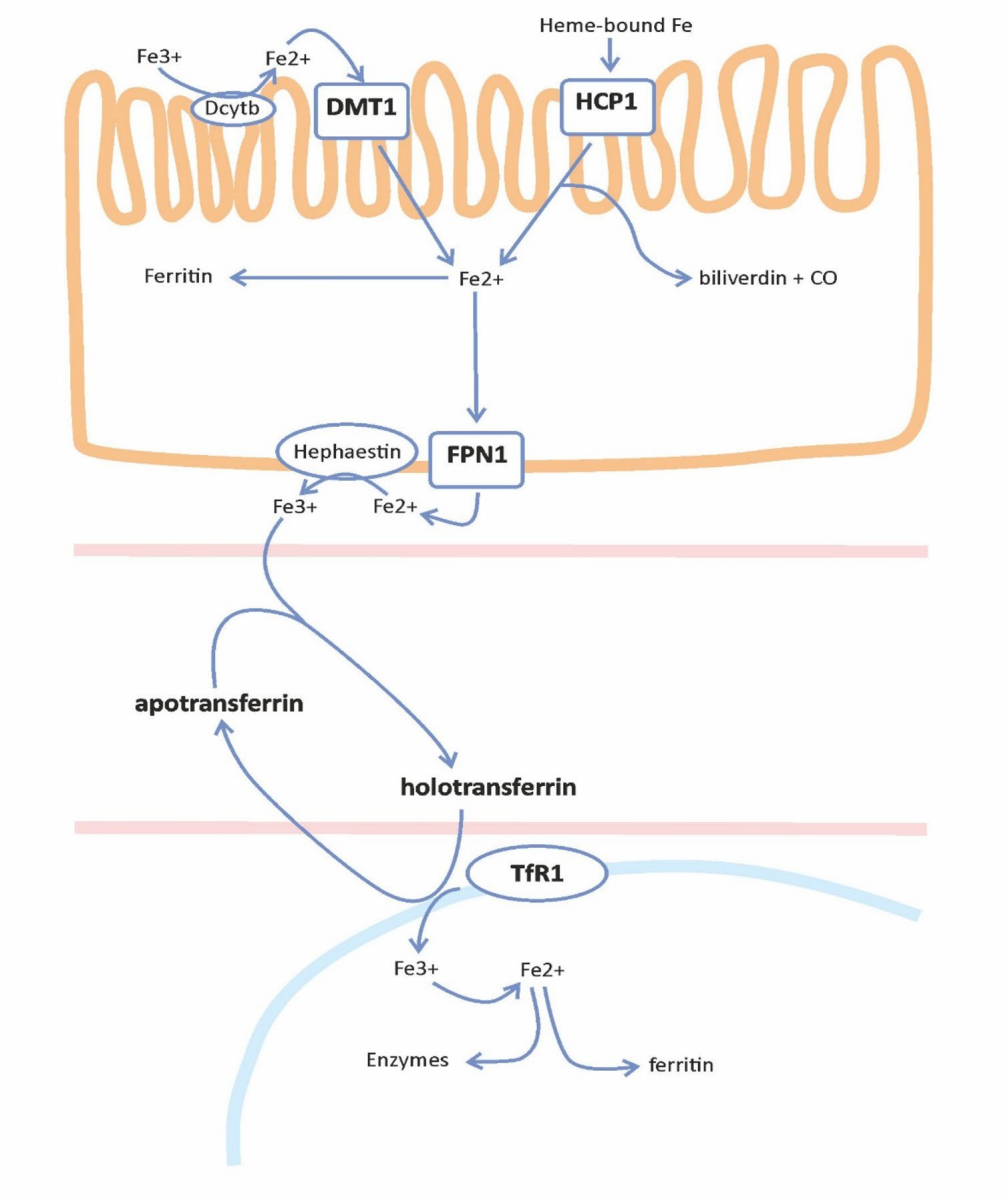
Iron absorption and metabolism. *Dcytb *duodenal cytochrome b-like ferrireductase, *DMT1 *divalent metal transporter 1, *HCP1* heme carrier protein 1, *FPN1* ferroportin, *TfR1* transferrin receptor 1

Iron is one of the essential elements in all living organisms. Approximately 71% of total body iron is found in hemoglobin and myoglobin in ferrous state (Fe^2+^). 25% is contained in storage proteins, ferritin and hemosiderin, in ferric state (Fe^3+^). The unique properties of iron, which can serve as both acceptor and donor of electrons, are responsible for its biologic functions but at the same time determine its toxicity. Excess iron may promote formation of reactive oxygen species (ROS) and lead to oxidative stress via damage to DNA, proteins and lipid membranes (Casu C Hepcidin agonists as therapeutic tools). As a heme cofactor, hemoglobin is responsible for transport of oxygen. Furthermore, iron is one of the compounds of catalase, peroxidase, cytochromes and other enzymes [6, 7]. Daily iron requirements amount to approximately 25–30 mg [8, 9], while iron loss (resulting from, e.g. menstruation, enterocyte and epidermal desquamation, with traces of iron also found in sweat and urine) constitutes 1–2 mg per day. There is no active mechanism that enables the elimination of iron. As a result, to maintain adequate iron balance, intestinal absorption of the element must cover daily iron loss. Healthy balanced diet provides approximately 10–20 mg of iron, of which 1–4 mg is absorbed [6, 10]. Absorption of ferrous iron (Fe^2+^) takes place in the apical enterocytes of the duodenum with the aid of the Divalent Metal Transporter 1 (DMT1), while heme can also be absorbed via Heme Carrier Protein 1 (HCP1) [4, 7]. Ferric iron (Fe^3+^) is not easily absorbed; therefore, the reduction of Fe^3+^ to Fe^2+^ is required. This process is enabled by duodenal cytochrome b-like ferrireductase enzyme (Dcytb). Absorbed iron can be stored in the enterocytes in ferritin-bound form (and usually lost as a result of enterocyte desquamation) or transported to the plasma via ferroportin (FPN1; also found in macrophages, hepatocytes and the placenta), where it binds to transferrin, which requires prior iron oxidation by hephaestin, a multicopper ferroxidase present on the basolateral membrane of the enterocyte. Transferrin, the essential iron-binding protein produced in the liver, can reversibly bind two ferric ions, thus changing its conformation to holotransferrin. Usually approximately 30–40% of transferrin molecules are saturated with iron, which means that the majority of transferrin in the plasma has conformation called apotransferrin and can buffer excess iron, if necessary. Circulating holotransferrin can be taken up by cells which have increased iron demands, e.g. erythrocyte precursors, via binding to transferrin receptor 1 (TfR1). Holotransferrin enters the cytoplasm by means of endocytosis and as a result of pH-associated change in conformation, releases iron ions. Ferric iron is once again reduced to Fe^2+^ and crosses endosomal barrier via DMT1 and is ready to be incorporated into various enzymes or storage proteins (Fig. 1).

## Role of hepcidin

Hepcidin, a 25-amino acid polypeptide discovered in 2000, is one of the key elements of systemic iron metabolism [11]. Hepcidin is a hormone produced predominantly in the hepatocytes and released into the plasma. It binds to ferroportin present in the cell membrane of enterocytes and macrophages and via tyrosine phosphorylation leads to internalization of ferroportin and eventually its degradation in the lysosomes. As a result, iron transport from the duodenum to the blood circulation is diminished, iron release from macrophages and hepatocytes is blocked and, consequently, iron recirculation is impaired and serum iron levels decrease [6]. Hepcidin production increases in response to iron overload, inflammation or infection, while its synthesis is diminished as a result of iron deficiency, increased erythropoiesis and hypoxia. Hepcidin expression is regulated by numerous proteins—bone morphogenetic protein-6 (BMP-6), hemojuvelin (HJV), human hemochromatosis protein (HFE), transferrin receptors TfR1 and TfR2, among others. They are influenced by both liver iron stores and circulatory iron in the form of iron-bound transferrin (holotransferrin) [12, 13]. Increased iron levels stimulate production of BMP-6, which binds to its receptor on the surface of hepatocyte and forms a complex with HJV (a membrane-bound co-receptor). This process induces SMAD (small mothers of decapentaplegic) phosphorylation pathway, leading to increased expression of hepcidin genes. HFE forms a complex with TfR and beta-2-microglobulin and TfR2, which in a not-yet-known fashion induces transcription of hepcidin genes. Mutations in the abovementioned proteins cause hereditary hemochromatosis, which manifests itself in hepcidin deficiency and iron overload [14]. Erythropoiesis stimulating factors affect hepcidin synthesis as well—for example, in the event of excessive erythropoiesis, erythropoietin (via binding with EPO receptor on the surface of hepatocytes) and growth differentiation factor 15 (GDF-15) decrease expression of hepcidin [15]. Furthermore, increased erythropoiesis is associated with elevated concentration of soluble transferrin receptor (sTfR), which is cleaved from transmembrane transferrin receptor expressed mainly in cells with high iron demands [15].

**Fig. 2:**
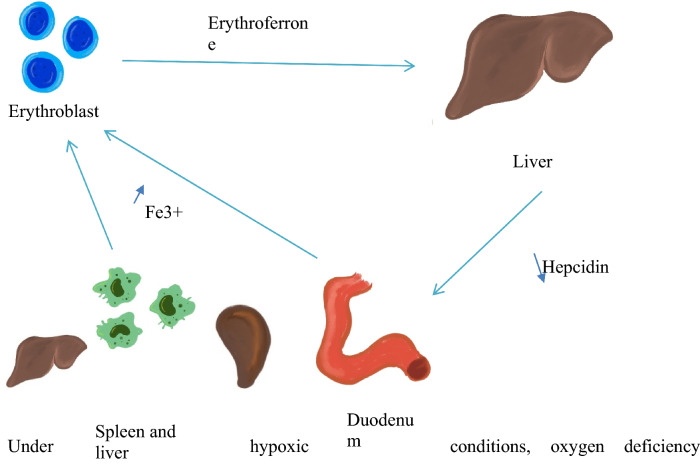
Increased erythroferrone production by erythroblasts suppresses hepcidin synthesis in the liver. Low hepcidin concentration increases iron availability for erythropoiesis by enhancing iron absorption in the duodenum and iron release from macrophages in the liver and the spleen

Recently, a hormone erythroferrone (ERFE) has been linked with erythropoiesis and iron balance. ERFE is synthesized in erythroblasts in response to increased erythropoiesis and it suppresses transcription of hepcidin in hepatocytes and thus increases iron availability in conditions associated with greater iron demand [16]. In murine models, ERFE deficiency is associated with mild hypochromic anemia and delayed hepcidin suppression following hemorrhage or EPO injection, while in certain conditions with ineffective erythropoiesis, such as β-thalassemia, ERFE concentrations were significantly increased [17] (Fig. 2).

Under hypoxic conditions, oxygen deficiency leads to diminished transcription of hepcidin genes (and consequently decreased production of hepcidin) via hypoxia-inducible factors (HIF-1α, HIF-2α) [18]. HIFs activate the expression of matriptase-2 (also known as the transmembrane protease, serine 6; TMPRSS6), which cleaves HJV from the HFE/TfR2/HJV complex, decreasing hepcidin synthesis [19]. Recently, it has been suggested that iron affects the release of fibroblast growth factor 23 (FGF-23)—a marker of increased risk of cardiovascular incidents, especially in CKD patients [20–23]. However, outcomes from clinical studies have so far been inconsistent; therefore, the impact of iron on FGF23 is unclear [24–26]. Finally, studies in humans revealed that hepcidin is one of the acute-phase proteins. It was proved that IL-1α, IL-1β, as well as IL-6 stimulate its expression [11, 27] via activation of the STAT3 transcription factor [28]—a mechanism responsible for iron restriction in the event of bacterial infection. Furthermore, mice with hepcidin antimicrobial peptide (HAMP) gene overexpression were affected by inflammation-related anemia of chronic diseases [29]. Conditions associated with inflammation (e.g. chronic kidney disease) lead to hepcidin-mediated iron sequestration in the reticuloendothelial cells and, consequently, decrease iron concentration in the system. At the same time, iron distribution becomes impaired; hence, iron availability for the synthesis of hemoglobin is reduced. As a result, anemia of inflammation (anemia of chronic diseases) develops [30]. In addition, in the setting of inflammation, patients can have high ferritin levels, low TSAT, and increased iron stores but still experience restricted erythropoiesis resulting from “reticuloendothelial blockade” [5]. Moreover, functional iron deficiency, a state of inadequate delivery of iron to the bone marrow in the setting of adequate iron stores, is caused by impaired iron mobilization (from the reticuloendothelial system [RES]) and/or increased bone marrow iron demand (as might be secondary to reduced red cell life span and/or erythropoiesis-stimulating agents [ESA] use) [31]. Proinflammatory cytokines are responsible for various processes typical of anemia of chronic diseases—they suppress erythropoiesis in the bone marrow, impair the production of erythropoietin (EPO) [31, 32] and increase the rate of erythrophagocytosis leading to shorter erythrocyte lifespan [6]. Moreover, in the event of chronic kidney disease the clearance of hepcidin is diminished and, as a result, its plasma concentration increases.

## Assessment of iron status

Understanding the dysregulation of iron metabolism is essential for the precise assessment, predicting treatment response as well as effective and safe treatment of anemia of chronic kidney disease. A number of biomarkers of iron status in chronic kidney disease have been used in clinical settings. However, many of them are influenced by renal failure alone and concomitant inflammation. Due to these confounding effects on the interpretation of most of biomarkers, the assessment of iron status in chronic kidney disease is still a challenge [5, 33, 34].

Serum iron, transferrin (Tf), total iron binding capacity (TIBC, calculated as Tf × 1389), transferrin saturation (TSAT, calculated serum iron/total iron binding capacity × 100) and serum ferritin are traditionally used in the evaluation of iron status and the diagnosis of iron deficiency anemia (IDA) [35]. In general population, decreased serum ferritin (< 15 ng/ml) and decreased TSAT (< 16%) are used for diagnosis of iron deficiency (ID) and iron deficiency anemia (IDA) in individuals without concomitant inflammation [35]. The international guidelines for the management of IDA in CKD use the same diagnostic tests; however, they recommend different cutoff levels of serum ferritin and TSAT for the diagnosis and initiation of iron supplementation. Some guidelines recommend higher cutoff levels of TSAT (≤ 30%) and serum ferritin (≤ 200–500 ng/ml) [36, 37], while others, lower TSAT < 20% and serum ferritin < 100 ng/ml [38–40]. The reason for the difference remains unclear, but at least in part, it may reflect the consideration of the influence of inflammation on iron metabolism disorders in CKD and the distinct prevalence of inflammation severity between patient populations in different countries. Regardless of the values adopted, the numerous limitations of these diagnostic tools in the assessment of iron stores in the storage and functional pools, and in predicting the response to treatment should be emphasized. The traditional cutoffs of TSAT at ≤ 20% and serum ferritin ≤ 100 ng/ml have low sensitivity in iron deficiency detection. In Stancu et al.’s [41] study, these indices identified only 17% patients with CKD stage 3–5 as iron deficient whereas 50% prove to be iron deficient based on bone marrow iron staining. Another limitation of these biomarkers is scant ability to differentiate between absolute and functional iron deficiency. It is assumed that low TSAT combined with normal or elevated serum ferritin level is diagnostic of functional iron deficiency [42]. However, if functional iron deficiency results of supply/demand mismatch, for example, during treatment with ESA, iron may transfer from transferrin faster than it can be mobilized from the iron stores, resulted in TSAT decrease [43].

The changes occurring in iron metabolism in CKD patients are different from those observed in iron deficiency in general population reflecting the effect of inflammation being a part of uremic state. With the progression of kidney disease, the production of transferrin in the liver is reduced, and in advanced stages of CKD, transferrin levels are reduced by 30% [44, 45]. As an acute-phase reactant, TIBC progressively decreases with kidney disease progression, and it leads to higher TSAT levels independent of iron status (13) and reduces its credibility as a measure of iron status and a threshold for initiating iron therapy in CKD patients [46]. In the meanwhile, in the majority of patients with stage 3–5 CKD, TSAT < 20% may correspond to serum iron levels below the lower limit and be indicative of iron deficiency [47]. It is postulated that more important parameter to assess iron status and prevent iron-limited erythropoiesis in CKD patients is iron concentration rather than TSAT. In recently published review, to avoid iron deficiency, target serum iron of 60 μg/dl was assumed, which corresponds to TSAT of 20% in CKD stage 3, and 22–25% in stage 4 or 5. It is established in clinical practice that serum iron is the more predictive index of iron sufficiency excluding iron-deficient erythropoiesis in hemodialysis patients [48].

Ferritin as an acute-phase reactant is frequently elevated in CKD patients irrespective of their iron stores [49]. Increased serum ferritin levels are the result of systemic inflammation and correlate positively with the severity of inflammation [50, 51]. Thus, the interpretation of serum ferritin is complicated by concomitant inflammation [35, 45]. Under minor inflammation, the specificity of low serum ferritin concentration of absolute iron deficiency diagnosis is high [35, 52], but if apparent inflammation is present, normal or elevated ferritin levels cannot exclude iron deficiency in CKD [35, 52]. Inflammation may also reduce the predictive value of serum ferritin for the response to iron supplementation. The baseline ferritin level may be predictive of the response to oral [53] and intravenous [54] iron treatment only in patients with minor inflammation expressed as low CRP level. Under concomitant inflammation, ferritin loses its predictive value of the response to iron therapy. Moreover, in highly inflamed patients, not only ferritin but also other biomarkers of iron metabolism (TSAT, CHr, or sTfR) lose their value in predicting treatment response [55–57]. In these patients, the values of CRP, but not indices of iron status may be predictive of the response to iron supplementation [55, 57], and there is no correction factor that could applied in estimation of iron stores depending on ferritin concentration [48].

The limitations of traditional biomarkers of iron metabolism and the response to treatment created the need to search for alternative diagnostic tools for iron management in CKD patients.

Soluble transferrin receptor (sTfR) is produced by proteolysis of the membrane transferrin receptor (TfR). Its release into circulation is increased in the setting of iron deficiency; hence, sTfR has been evaluated as a potential biomarker of iron deficiency. Soluble TfR is not an acute-phase reactant and is less influenced by inflammation than other iron metabolism indices [58]. The serum concentration of sTfR is increased in hemodialysis patients with iron deficiency and correlate inversely with iron available for erythropoiesis; however, it is not able to detect occult iron deficiency [42, 59, 60]. Unfortunately, the interpretation is confounded by the use of ESA [35, 45], and appears to represent erythropoietic activity rather than iron deficiency [42, 59, 60]. The index of sTfR to log_10_ ferritin has better than TSAT and ferritin predictive value of iron supplementation responsiveness in hemodialysis patients [61]. The limitations of widespread measurement of sTfR include not-established standard cutoffs, costs and availability in the laboratory.

Other biomarkers of iron status include reticulocyte hemoglobin content (CHr) and percentage of hypochromic red blood cells (%Hypo). CHr provides an expression of iron availability for erythropoiesis within 3–4 days [35, 45] and CHr < 27.2 pg is diagnostic for iron deficiency [35]. %Hypo measures the concentration of hemoglobin in red blood cells (RBC), which reflects absolute amount of hemoglobin and the RBC size [35, 45] and serves as a sensitive marker of iron deficiency [35, 45] and iron status changes in the long-term assessment [35, 45]. Both these biomarkers are influenced by inflammation [62, 63]. Nevertheless, CHr and %Hypo have, compared to TSAT and serum ferritin, better sensitivity and specificity to predict iron deficiency in late stages of CKD [48]. CHr and %Hypo are predictive of iron responsiveness [64, 65] with at least similar test accuracy compared with traditional biomarkers in predicting hemoglobin increase to intravenous iron administration [66]. It needs to be highlighted that during iron supplementation the temporal changes of CHr and %Hypo differs—CHr can normalize within 2–3 days, whereas %Hypo can take even months [48]. Unfortunately, both measurements are limited by testing requirements. %Hypo must be tested on fresh blood samples (within 6 h) and CHr is time sensitive due to the maturation of erythrocytes [42].

Hepcidin, given its central role in iron metabolism regulation, has been evaluated as a biomarker of iron status and iron responsiveness in CKD patients. Many studies have confirmed increased hepcidin levels in CKD patients [67–69] Serum hepcidin levels have the strongest correlation with serum ferritin, TSAT and sTfR [67, 70–72] and are influenced by inflammation [67, 72, 73] iron and ESA administration [67, 71, 72, 74] and dialysis clearance [68, 69, 75]. Due to a significant intra-individual variability, the short-term measurement of serum hepcidin is not useful as a biomarker of iron status in CKD patients [76, 77]. And the interpretation of the serum hepcidin level must take into account all confounding factors. Hepcidin is not a good predictor of the response to iron supplementation in dialysis [57] and non-dialysis-dependent patients [78].

Soluble hemojuvelin (sHJV) has been explored as biomarker of iron status in patients without [79], and with chronic kidney disease [80, 81]. Opposite to cell surface HJV, soluble HJV may act as an inhibitor of BMP signaling and restrain hepcidin expression [82]. Some studies revealed that sHJV may be increased in iron deficiency and decreased during iron administration [82–84], suggesting that sHJV may be a diagnostic marker of iron status. One important concern in soluble HJV assessment is assay validity [81, 82, 85], and future studies are needed to establish sHJV value as biomarker of iron status and response to therapy.

Growth differentiation factor 15 (GDF15), secreted by matured erythroblasts, is involved in hepcidin metabolism and as such is potentially involved in iron metabolism [86]. However, available data on the role of GDF15 as the marker of iron status are scarce. In fact, one study suggested that GDF15 is increased in iron deficiency [87]; the other did not confirm it [88]. Moreover, serum GDF15 levels may be influenced by kidney disease, malnutrition and inflammation [89] complicating its usefulness as an iron status biomarker.

Plasma neutrophil gelatinase-associated lipocalin (NGAL) is known as a predictor of kidney disease progression and marker of inflammation [90, 91]. In addition, NGAL influences iron sequestration; however, the way that NGAL influences the iron balance depends on its form. The bound form of NGAL decreases and free form of NGAL increases the level of serum iron. Few studies evaluated the usefulness of NGAL as a biomarker of iron stores in CKD patients, suggesting its good specificity and sensitivity in the detection of decreased iron stores [92, 93]; however, future studies are needed to establish the role as a biomarker of iron status.

In summary, we conducted a search in Medline, PubMed, and Embase using the keywords: iron, biomarkers, kidney failure, CKD, dialysis, hemodialysis, peritoneal dialysis. As described in the Preferred Reporting Items for Systematic reviews and Meta-Analyses (PRISMA) group [94]. We limited our search to adult patients and publications in English and Polish till 2020. We found 541 articles, but only 102 articles were analyzed due to lack of information about full data, and availability of abstracts only or duplication. The available data were very limited due to a high degree of heterogeneity. Taking into account the drawbacks and sometimes limited data on the utility of alternative biomarkers of iron status in chronic kidney disease, the traditional biomarkers still remain the hallmarks of the assessment of iron metabolism and responsiveness to iron therapy in this patient population.

## Therapeutic strategies

Therapeutic approach should begin with diagnosis and elimination of the underlying condition responsible for iron deficiency. Iron supplementation is the next step. Initiation of iron in CKD patients with anemia should be based on preexisting iron stores and the target Hb level that is desired. Even though oral iron is generally considered sufficient in CKD patients not on dialysis and PD patients, intravenous form is the preferred route, especially in hemodialysis patients. Oral iron is associated with poor intestinal absorption and adverse event-related (mainly gastrointestinal) low adherence to therapy [94]. It needs to be emphasized that the goal of treatment with iron is not to increase Hb levels to the normal range but to reduce the risk of development of severe anemia and associated complications and to minimize the need for blood transfusions [4]. ESA therapy is generally initiated in ESRD patients with replete iron stores (i.e. TSAT > 30% and/or ferritin > 500 mcg/l) whose Hb levels are below 10 g/dl [95]. Failure to increase Hb concentration after the first month of ESA treatment is defined by KDIGO as ESA hyporesponsiveness—a poor prognostic factor in terms of patient mortality [36]. There are several factors responsible for ESA hyporesponsiveness—one of them is inflammation. A new group of oral agents known as HIF prolyl hydroxylase-inhibitors has been developed to improve CKD-associated anemia. The beneficial effect of HIF stabilizers on hemoglobin levels has been observed regardless of the patient’s iron stores and inflammatory status [17]. However, the long-term safety of these novel agents, especially regarding potential risk of tumorigenesis and worsening of proliferative diabetic retinopathy, has yet to be established [17, 96]. Additionally, the increased usage of intravenous iron in hemodialysis patients during recent years has led to increasing concern over the potential development of iron overload [97, 98]. Recently, we reported that a substantial number of hemodialysis patients have positive labile plasma iron after intravenous iron administration, which positively correlated with laboratory parameters that are currently in routine clinical use for detecting iron overload and with higher intravenous iron dose [99]. Thus, we suggest to perform further studies to establish the clinical implications of labile plasma iron, a component of nontransferrin-bound iron which may be a more accurate indicator of impending iron overload monitoring in hemodialysis patients.

## Conclusion

CKD patients tend to have subclinical inflammatory-related immune activation. The pathogenesis of chronic inflammation in CKD is still not fully understood, yet the proposed underlying factors include oxidative stress, cellular senescence, hypoxia, exogenous factors (such as dialyzer membrane or central venous catheter), immune dysfunction, gut dysbiosis and retention of uremic toxins [100]. Inflammatory blockade is associated with resistance to erythropoietin despite iron availability, which is more clearly understood now that the role of hepcidin in iron metabolism has been identified. Studies conducted so far revealed that serum ERFE concentration increases in response to ESA treatment in CKD patients, while the correlation between ERFE and hepcidin remains unclear [101]. Despite having classical iron biomarkers, we still looking for new ones to improve our diagnostic and predictive tools. There is an area of uncertainty regarding diagnostic utility of both ERFE and hepcidin in ESRD patients [102]. Higher concentration of hepcidin in oligoanuric patients may reflect decreased renal clearance. Furthermore, the hepcidin-lowering effect of ERFE in ESRD patients treated with ESAs may be blunted by underlying inflammation and concomitant iron treatment. Up to date, we have no cost-effective analytical tests to assess iron metabolism in patients with CKD. Therefore, future studies should validate the use of ERFE as a biomarker of erythropoiesis and predictor of response to iron and ESA therapy in dialysis-dependent patients.
